# Asante Calcium Green and Asante Calcium Red—Novel Calcium Indicators for Two-Photon Fluorescence Lifetime Imaging

**DOI:** 10.1371/journal.pone.0105334

**Published:** 2014-08-20

**Authors:** Karolina Jahn, Carsten Hille

**Affiliations:** Department of Physical Chemistry/ALS ComBi, University of Potsdam, Potsdam, Germany; University of Hull, United Kingdom

## Abstract

For a comprehensive understanding of cellular processes and potential dysfunctions therein, an analysis of the ubiquitous intracellular second messenger calcium is of particular interest. This study examined the suitability of the novel Ca^2+^-sensitive fluorescent dyes Asante Calcium Red (ACR) and Asante Calcium Green (ACG) for two-photon (2P)-excited time-resolved fluorescence measurements. Both dyes displayed sufficient 2P fluorescence excitation in a range of 720–900 nm. *In vitro*, ACR and ACG exhibited a biexponential fluorescence decay behavior and the two decay time components in the ns-range could be attributed to the Ca^2+^-free and Ca^2+^-bound dye species. The amplitude-weighted average fluorescence decay time changed in a Ca^2+^-dependent way, unraveling *in vitro* dissociation constants *K*_D_ of 114 nM and 15 nM for ACR and ACG, respectively. In the presence of bovine serum albumin, the absorption and steady-state fluorescence behavior of ACR was altered and its biexponential fluorescence decay showed about 5-times longer decay time components indicating dye-protein interactions. Since no ester derivative of ACG was commercially available, only ACR was evaluated for 2P-excited fluorescence lifetime imaging microscopy (2P-FLIM) in living cells of American cockroach salivary glands. In living cells, ACR also exhibited a biexponential fluorescence decay with clearly resolvable short (0.56 ns) and long (2.44 ns) decay time components attributable to the Ca^2+^-free and Ca^2+^-bound ACR species. From the amplitude-weighted average fluorescence decay times, an *in situ K*_D_ of 180 nM was determined. Thus, quantitative [Ca^2+^]_i_ recordings were realized, unraveling a reversible dopamine-induced [Ca^2+^]_i_ elevation from 21 nM to 590 nM in salivary duct cells. It was concluded that ACR is a promising new Ca^2+^ indicator dye for 2P-FLIM recordings applicable in diverse biological systems.

## Introduction

The basis of all cellular processes and activities is the precise regulation of intracellular ion homeostasis. Calcium ions (Ca^2+^) serve as ubiquitous intracellular second messengers and play a pivotal role in numerous processes, such as neuronal signaling, fluid secretion, exocytosis or contraction. Regulation of the intracellular Ca^2+^ concentration ([Ca^2+^]_i_) is maintained by a variety of channels, exchangers and pumps located in the plasma membrane or in internal membranes. Thus, this Ca^2+^ signaling toolkit results in an appropriate stimulus-induced temporal and spatial intracellular Ca^2+^ pattern [1], [2], [3]. Hence, quantification of [Ca^2+^]_i_ and its dynamics is crucial for a better understanding of physiological processes as well as dysfunctions.

Due to the variety and multidimensional characteristics of Ca^2+^-sensitive fluorescent dyes, highly sensitive and non-invasive fluorescence microscopy is a versatile tool for studying [Ca^2+^]_i_ [4], [5], [6], [7]. In general, two families of fluorescent Ca^2+^ indicators can be considered, whose spectroscopic properties change differently upon Ca^2+^ binding. Genetically encoded fluorescent proteins are based on variants of the green fluorescent protein [8]. In contrast, a broad range of organic Ca^2+^ indicators with different spectral properties have been developed in the last decades [4], [7]. One of the most important parameters of a Ca^2+^ indicator is its Ca^2+^ binding affinity represented by the dissociation constant *K*_D_. The measureable range of Ca^2+^ concentrations is typically restricted to 0.1×*K*_D_–10×*K*_D_. Thus, depending on the particular biological system, high-affinity Ca^2+^ indicators with low *K*_D_ values or low-affinity Ca^2+^ indicators with high *K*_D_ values are preferred [7]. However, for reliable [Ca^2+^]_i_ quantification it is crucial to empirically determine *K*_D_ within the particular biological system, as it strongly depends on temperature, pH, viscosity, ionic strength or protein binding [7]. Most commonly, the fluorescence intensity *I*_F_ as a Ca^2+^-dependent parameter is recorded. However, fluorescence intensity-based measurements are sometimes prone to distinct drawbacks, as the fluorescence intensity depends on the dye concentration [9]. Due to dye leakage, dye bleaching and changes in the cellular volume, the dye concentration can alternate within the imaging process, making Ca^2+^ quantification more difficult. Ratiometric imaging is one method to obtain more trustworthy quantitative data. Here, fluorescent Ca^2+^ indicators are used, which exhibit a shift in their excitation or emission spectra upon Ca^2+^ binding. Thus, depending on the indicator type, two fluorescence images are recorded using two excitation or emission wavelengths. The calculated ratio allows a correction of varying indicators' fluorescence intensity and is therefore almost unaffected by changes to the indicator concentration and optical pathway [9]. However, there is also evidence of insufficient correction when using the ratiometric approach, indicating the necessity of calibration measurements for every individual experiment [10]. Moreover, a ratiometric approach using two excitation wavelengths is difficult to implement in multiphoton microscopy.

Another approach, circumventing the above-mentioned pitfalls, is fluorescence lifetime imaging microscopy (FLIM), which can also be combined with two-photon (2P) excitation [11], [12]. Here, the fluorescence decay time τ of the Ca^2+^ indicator is the recording parameter. It encompasses the average time an excited molecule remains in the excited state before spontaneous emission occurs. The fluorescence decay time, which is mostly independent of dye concentration, allows reliable quantitative ion recordings. Two main techniques provide access to the fluorescence decay time. In the frequency-domain techniques, the fluorescence decay time is calculated indirectly from the phase shift and demodulation of the fluorescence emission relative to the excitation light. In contrast, time-domain techniques use a pulsed excitation laser and record the fluorescence decay function directly [12]. By now, several Ca^2+^-sensitive dyes, which were originally synthesized for fluorescence intensity-based measurements, have been proven for FLIM recordings, such as Calcium Green, Quin-2 and Oregon Green BAPTA-1 [6], [5], [13], [14]. A prerequisite for fluorescence lifetime-based measurements is a change in the Ca^2+^ indicator's fluorescence quantum yield upon Ca^2+^ binding [15]. Consequently, indicator dyes are not *a priori* suitable for FLIM recordings, and comprehensive characterization of each indicator dye is required. Asante Calcium Green (ACG) and Asante Calcium Red (ACR) are newly developed Ca^2+^-sensitive fluorescent dyes (Teflabs Inc., Austin, USA). Due to their improved spectral properties with greatly enhanced brightness and a large Stokes shift allowing single-wavelength and ratiometric imaging, these dyes seem to be promising new Ca^2+^ indicators [16]. In this study, ACG and ACR were characterized for time-resolved fluorescence measurements *in vitro*. Moreover, the suitability of ACR for 2P-FLIM recordings was successfully demonstrated in living tissue. Here, salivary glands of the American cockroach *Periplaneta americana* were used, representing a well-established model system for studying biogenic amine-regulated epithelial ion transport [17], [18].

## Materials and Methods

### Chemicals and solutions

For *in vitro* measurements, the Calcium Calibration Buffer Kit #1 (Life Technologies, Darmstadt, Germany) (pH 7.2, ϑ = 22°C, *I* = 0.1) was used to prepare 11 solutions of varying Ca^2+^ concentrations [Ca^2+^]_free_ (in nM: 0, 20, 40, 70, 100, 150, 230, 360, 620, 1390, 39660). For investigating protein-dye interaction, Ca^2+^-free and Ca^2+^-saturated buffer solutions were prepared containing 18 mM NaCl, 2 mM MgCl_2_·6 H_2_O, 40 mM KCl, 80 mM K gluconate, 5 mM HEPES and 1% (m/v) bovine serum albumin (BSA) (Sigma Aldrich, Deisenhofen, Germany). Additionally, the Ca^2+^-free buffer solution contained 2 mM EGTA and the Ca^2+^-saturated buffer solution 2 mM Ca(NO_3_)_2_. The pH was adjusted to pH 7.3 with 1 N HCl.

The K^+^-salts of the Ca^2+^-sensitive dyes Asante Calcium Red (ACR 25 µg, Teflabs Inc., Austin, USA) and Asante Calcium Green (ACG 25 µg, Teflabs Inc., Austin, USA) were dissolved in 50 µL double-distilled water to obtain 0.5 mM and 0.4 mM stock solutions, respectively. These stocks were then diluted in the Ca^2+^ buffer solutions, reaching final concentrations of [ACR]  = 2.5 µM and [ACG]  = 0.9 µM, respectively.

Cockroach physiological saline contained 160 mM NaCl, 10 mM KCl, 2 mM MgCl_2_·6 H_2_O, 2 mM CaCl_2_·2 H_2_O, 10 mM glucose and 10 mM Tris, pH 7.4. According to [4], a Ca^2+^-free stock solution of 0.5 M K_2_H_2_EGTA and a Ca^2+^-saturated stock solution of 1 M K_2_CaEGTA were prepared by potentiometric back titration for *in situ* calibration experiments. These stocks were then diluted in a buffer solution (160 mM NaCl and 10 mM Tris) to the final concentration of 10 mM K_2_H_2_EGTA and 10 mM K_2_CaEGTA calibration buffer solutions (pH 7.4, adjusted with HCl). By mixing K_2_H_2_EGTA and K_2_CaEGTA, various free Ca^2+^ concentrations [Ca^2+^]_free_ could be obtained according to Equation (1).
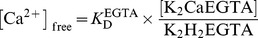
(1)


The given EGTA dissociation constant 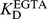 depends on temperature, ionic strength and pH, and under recent conditions (pH 7.4, ϑ = 20°C, *I* = 0.16) it could be determined through a Beers fitting procedure at 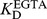  = 68.1 nM [19].

For *in situ* calibration experiments, the non-ionic surfactant Triton X-100 (Sigma Aldrich, Deisenhofen, Germany) was used to equilibrate defined extracellular and intracellular Ca^2+^ concentrations [20], [21]. Hence, the salivary glands were continuously perfused with calibration buffer solutions containing a defined [Ca^2+^]_free_ (in nM: 0, 20, 80, 460, 790, 2360, 33960, 680450) and 0.1% Triton X-100 (v/v). A 10 mM stock solution of dopamine (Sigma Aldrich, Deisenhofen, Germany) in double-distilled water was prepared daily and dissolved in physiological saline immediately before an experiment to a final dopamine concentration of 1 µM. The acetoxymethyl (AM)-ester of ACR (50 µg, Teflabs Inc., Austin, USA) was diluted in 27 µL Pluronic F-127 (20%-solution in DMSO, Sigma Aldrich, Deisenhofen, Germany), divided into 1 µL aliquots and stored at −20°C. The aliquots were dissolved in hypotonic physiological saline (75% physiological saline +25% water) immediately before an experiment to the final dye concentration of 5 µM.

### Absorption and fluorescence measurements

Absorption measurements were performed with a Lambda 750 UV/VIS spectrometer (Perkin Elmer, Waltham, USA). To determine the absorption coefficients, absorption spectra in Ca^2+^-free and Ca^2+^-saturated buffer solutions were recorded. The dye concentration varied from 1.7 µM to 12.5 µM for ACR and from 0.9 µM to 1.8 µM for ACG. Fluorescence quantum yields of the Ca^2+^-saturated dye forms were determined absolutely with the C 9929 integration sphere system (Hamamatsu, Hamamatsu City, Japan). Since the fluorescence quantum yields of the Ca^2+^-free dye forms were below the detection limit of this system (Φ_F_ <0.01), fluorescence quantum yields of these dye forms were determined relative to the respective Ca^2+^-saturated form as a fluorescent reference [25], [26]. Steady-state fluorescence spectra were recorded with FluoroMax 4 (Horiba, Kyoto, Japan). For time-resolved fluorescence measurements in the BSA-buffer, ACR was excited by a supercontinuum source (SC-400-PP, Fianium, Southhampton, UK) operating at λ_ex_ = 550 nm with a repetition rate of 20 MHz and a pulse width of ∼30 ps. The laser beam was fiber-guided towards the fluorescence lifetime spectrometer FL920 (Edinburgh Instruments, Edinburgh, UK), where the emitted fluorescence was detected by a multichannel plate (ELDY EM1-123/300, EuroPhoton, Berlin, Germany) in the time-correlated single photon counting (TCSPC) mode.

### 2P fluorescence excitation spectra

2P fluorescence excitation action cross-sections Φ_F_σ_2_ were determined from relative measurements using the well-characterized 2P-reference rhodamine B in methanol [22], [23]. Rhodamine B concentrations were adjusted for the respective samples and controlled by absorption spectra if possible. Thus, for 2.5 µM ACR, the rhodamine B concentration was adjusted to 3 nM and 0.1 µM in Ca^2+^-free and Ca^2+^-saturated conditions, respectively. For 0.9 µM ACG, the rhodamine B concentration was adjusted to 10 nM and 5.0 µM in Ca^2+^-free and Ca^2+^-saturated conditions, respectively. The Φ_F_σ_2_ values with 10^−50^ cm^4^ s/photon  = 1 GM were calculated according to Equation (2)



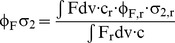
(2)where *c* is the dye concentration, Φ_F_ the fluorescence quantum yield, and 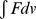 the integral of the 2P-fluorescence emission spectra [22], [23]. The subscript *r* indicates the spectroscopic parameters of the 2P-reference rhodamine B. 2P-excitaiton was carried out by a Ti:Sa laser system (Tsunami 3960; Spectra Physics, Mountain View, USA) tuning the wavelength between λ_ex,2P_ = 720 nm–900 nm, with a 82 MHz repetition rate and pulse width of ∼80 fs. The laser beam was coupled to the fluorescence lifetime spectrometer FL920 (Edinburgh Instruments, Edinburgh, UK) and was focused by a lens on the quartz cuvette. The fluorescence emission was detected with a photomultiplier (S300 blue-sensitive R1527, Hamamatsu, Hamamatsu City, Japan) in a spectral range of 400 nm–700 nm in 1 nm steps. The obtained emission spectra were integrated, unraveling 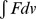. The stability of the average laser power was controlled between the measurements with a power meter (Fieldmaster LM10 HTD with a detection range of 10 mW to 10 W). The error of the determined σ_2_ values is estimated at 10%.

### Tissue preparation

A colony of the American cockroach *P. americana* (L.) was reared at 27°C under a light/dark cycle of 12 h: 12 h at the Department of Animal Physiology (University of Potsdam). The animals had free access to food and water. Only male adults aged between 4–6 weeks were used for experiments. Salivary glands were dissected in physiological saline as described previously [24]. Small lobes consisting of several acini with their corresponding branched duct system were examined.

### 2P-FLIM recordings

Salivary gland lobes were incubated for 60 min in hypotonic saline containing 5.1 µM ACR/AM. After dye loading, the gland lobes were transferred to physiological saline for at least 10 min, then fixed on a coverslip coated with the tissue adhesive Vectabond Reagent (Enzo Life Science, Lörrach, Germany) and mounted on a microscope stage. During the experiment, the gland lobes were continuously perfused with physiological saline at a flow rate of 2 ml/min.

MicroTime200 (PicoQuant, Berlin, Germany) was used for time-resolved fluorescence measurements of *in vitro* (Calcium Calibration Buffer Kit #1) and *in situ* imaging. The system includes an inverted microscope (IX71, Olympus, Hamburg, Germany) equipped with an Olympus PlanApo ×100/NA1.4 oil-immersion objective. A mode-locked fs-fiber laser (C-Fiber A 780; MenloSystems, Martinsried, Germany) was used as a 2P-excitation source, operating at λ_ex,2P_ = 780 nm with a repetition rate of 50 MHz and a ∼90 fs pulse width. The laser beam was guided towards the objective via the microscope side port using a dichroic mirror (2P-dichroic 725; Chroma, Fürstenfeldbruck, Germany). Fluorescence was guided through a 100 µm pinhole and was detected by a single-photon avalanche diode (SPCM-AQR-13; Perkin Elmer, Waltham, USA). For rejection of the excitation light, two short pass filters were used (1×SP420-680 OD2, Baader, Mammendorf, Germany; 1×SP400-680 OD4, Edmund Optics, Karlsruhe, Germany). Time-resolved fluorescence was performed in the TCSPC-mode by using a PicoHarp300 (PicoQuant, Berlin, Germany) with a time resolution of 8 ps. Laser power was adjusted to achieve average photon counting rates of <10^5^ photon/s and peak rates close to 10^6^ photons/s, thus below the maximum counting rate allowed by the TCSPC electronics to avoid pulse pile-up. The image acquisition occurred by raster scanning the objective using a *xy*-piezo-positioner (Physik Instrumente, Karlsruhe, Germany). Thereby, full-frame images of 80 µm×80 µm (150 pixel ×150 pixel) were acquired in around 30 s with a pixel dwell time of 0.6 ms. Data acquisition and analysis were performed by the SymPhoTime software version 5.3.2.2 (PicoQuant, Berlin, Germany). All photons collected in a distinct region of interest (ROI) were used to form a global histogram. The obtained decay curves were analyzed by a deconvolution fitting routine, whereby the quality of the fit was estimated by reduced 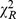 values and randomly distributed residuals. The required instrument response function (IRF) was measured every day by recording the backscattered excitation light [21]. Its full width at half-maximum (FWHM) was calculated at 220±2 ps (N = 21). Global multiexponential fluorescence lifetime analyses of several fluorescence decay curves with linked decay time components were carried out using FAST Software 2.13 (Edinburgh Instruments Ltd., Livingston, UK). In this case, two types of 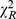 values were calculated. An individual 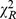 value defines the quality of fit to an individual decay curve within the global analysis. A global 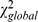 value characterizes the quality of fit for the entire set of data and was calculated as the root mean square of all individual 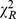 values. Based on the fluorescence lifetime analysis, every pixel in the image was then treated in SymPhoTime in the same way, but this time with a maximum likelihood estimator resulting in false color-coded FLIM images. In addition, fluorescence intensity images could be calculated by integrating all detected photons in every pixel, thereby ignoring the temporal information.

### Data analyses

Statistically analyzed sets of data were distributed normally (D'Agostino-Pearson normality test). Thus, the data sets were further analyzed by repeated-measures ANOVA and Holm-Sidak's multiple comparison post-hoc tests. Differences were considered statistically significant if *P*<0.05. Statistical analyses were performed using GraphPad Prism 4.01 (GraphPad Software, San Diego, USA). Graphical presentation was carried out using Origin 8 (OriginLab, Northampton, USA). Data are presented as the mean ± the standard error of the mean (SEM). The dissociation constants *K*_D_ were calculated by fitting the data points to Equation (3)


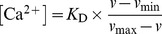
 or rather



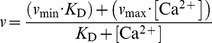
(3)where *v* corresponds to fluorescence intensity and fluorescence decay time, respectively [6]. The subscript *min* refers to Ca^2+^-free buffer conditions and the subscript *max* to Ca^2+^-saturated buffer conditions.

## Results and Discussion

### *In vitro* steady-state absorption and fluorescence measurements

**Figure 1:**
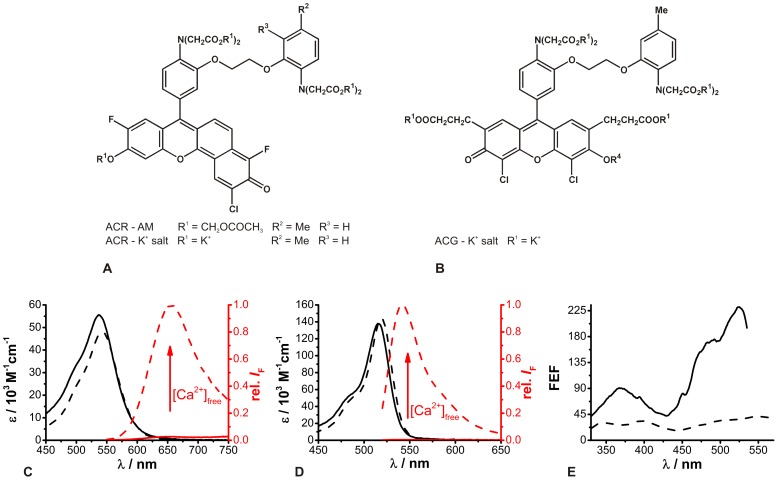
Steady-state absorption and fluorescence measurements for ACR and ACG. Chemical structures of (A) ACR and (B) ACG [16], [27]. Absorption (black) and relative fluorescence (red) spectra in Ca^2+^-free (solid line, [Ca^2+^]_free_  = 0 nM) and Ca^2+^-saturated (dashed line, [Ca^2+^]_free_  = 40 µM) buffers. (C) ACR (c = 2.5 µM, λ_ex_ = 540 nm). (D) ACG (c = 0.9 µM, λ_ex_ = 517 nm). (E) Fluorescence enhancement factor (FEF) of ACR (dashed line) and ACG (solid line) as a function of excitation wavelength. FEF is the ratio of the fluorescence intensities of the excitation spectra under Ca^2+^-saturated conditions and Ca^2+^-free conditions. The curves are the smoothed means of two measurements.

ACR is composed of a Ca^2+^ chelating BAPTA ligand and seminaphtofluorescein as the fluorescent backbone, as published recently (Figure 1A) [16]. According to a rise in [Ca^2+^]_free_, the absorption maximum of ACR shifted from 537 nm (Ca^2+^-free) to 542 nm (Ca^2+^-saturated) (Figure 1C). Concurrently, the molar absorption coefficient decreased from (55.0±1.3) 10^3^ M ^−1^ cm ^−1^ (N = 3, λ_abs_ = 540 nm) to (47.8±0.8) 10^3^ M ^−1^ cm ^−1^ (N = 3, λ_abs_ = 540 nm). These absorption coefficients fit well with the published data [16]. ACR exhibited a broad emission band around 654 nm, whose fluorescence intensity was elevated due to increasing [Ca^2+^]_free_ (Figure 1C). The fluorescence quantum yield of Ca^2+^-bound ACR at λ_ex_ = 515 nm was determined absolutely at Φ_F_ = 0.06±0.01 (N = 6), whereas it was estimated for the Ca^2+^-free ACR relatively at Φ_F_ = 0.0017 as its fluorescence quantum yield was below the limit of detection to be determined by absolute measurements. Here, ACR in a Ca^2+^-saturated buffer was used as the fluorescent reference [25], [26]. Tuning the excitation wavelength to shorter wavelengths (e.g. λ_ex_ = 488 nm), a further fluorescence emission band around 525 nm appeared (Figure S1). However, the fluorescence intensity of this emission band was almost independent of [Ca^2+^]_free_ changes. Thus, ACR can be used as an emission-ratiometric dye by recording fluorescence at a Ca^2+^-dependent and a Ca^2+^-independent emission wavelength, which is particularly useful in terms of reliable Ca^2+^ quantification [7].

Analog to ACR, ACG is composed of a Ca^2+^ chelating BAPTA ligand and a fluorescein derivative as the fluorescent backbone (Figure 1B) [27]. The absorption maximum of ACG shifted from 515 nm (Ca^2+^-free) to 519 nm (Ca^2+^-saturated) (Figure 1D), whereas the molar absorption coefficient also increased from (130±2.0) 10^3^ M ^−1^ cm ^−1^ (N = 3, λ_abs_ = 520 nm) to (144±1.6) 10^3^ M ^−1^ cm ^−1^ (N = 3, λ_abs_ = 520 nm). Increasing [Ca^2+^]_free_ caused a rise in the fluorescence intensity with an emission maximum at 542 nm. The fluorescence quantum yield of Ca^2+^-bound ACG at λ_ex_ = 515 nm was determined absolutely at Φ_F_ = 0.42±0.01 (N = 6), whereas it was estimated for the Ca^2+^-free ACG-relatively at Φ_F_ = 0.0020 using ACG in a Ca^2+^-saturated buffer as the fluorescent reference. Presumably, for ACG as well as ACR, a photoinduced electron transfer is supposed to be the mechanism for changing fluorescence characteristics. Thereby, the Ca^2+^ complexation by BAPTA moiety blocks the electron transfer from the electron donor BAPTA to the fluorophore, which acts as an electron acceptor. Here, the fluorescence intensity also increases [28]. ACG and ACR absorb and emit in the visible spectral range. This fact is quite advantageous for biological applications, because side effects such as cell toxicity and autofluorescence occurring with excitation in the UV spectral range are minimized. A measure for the dynamic sensor range is the fluorescence enhancement factor (FEF) as a ratio of the fluorescence intensities in Ca^2+^-saturated and Ca^2+^-free buffers. Thereby, both dyes displayed a dependency of the FEF and the chosen excitation wavelength (Figure 1E). A maximum FEF was achieved for ACR (41-fold) at λ_ex_ = 548 nm and for ACG (231-fold) at λ_ex_ = 525 nm. The excitation-dependent fluorescence enhancement for ACR has been described recently [16]. For both dyes, the obtained FEF values correspond well with literature data [16], [29].

From steady-state measurements in commercial Calcium Calibration Buffer Kit #1, the dissociation constants *K*_D,i_ were calculated using Equation (3). In the case of ACG, the received *K*_D,i_ of 265 nM±33 nM (N = 4) is nearly twice as high as that of the manufacturer (135 nM) [29]. However, due to missing data on the experimental conditions, a comparison of the data is not feasible, as varying ionic strength, viscosity and pH might have an impact on the determined *K*_D,i_. Depending on the excitation wavelength, ACR can be used in either single wavelength (λ_ex_ = 540 nm, λ_em_ = 655 nm) or dual wavelength (λ_ex_ = 488 nm, λ_em_ = 655 nm/525 nm) mode [16]. The fluorescence intensity of the emission band at 525 nm is almost independent of [Ca^2+^]_free_. In this case, the ratio of the fluorescence intensities at 655 nm and 525 nm were used to calculate the corresponding *K*_D,i_ = 415 nM±43 nM (N = 4). In the single wavelength mode of ACR, a *K*_D,i_ = 463 nM±59 nM (N = 4) was calculated. In both cases, experimental data agree well with the manufacturer's data (*K*_D,i_ = 400 nM) [29]. Concluded from the determined *K*_D,i_ values, ACG and ACR are both high-affinity Ca^2+^ indicators [7].

### 2P fluorescence excitation spectra

**Figure 2:**
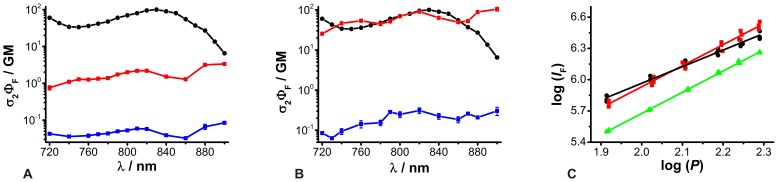
2P fluorescence excitation spectra for ACR and ACG. Logarithmic plot of 2P fluorescence excitation action cross-sections Φ_F_σ_2_ as a function of excitation wavelength. (A) ACR (means ± SEM, N = 6). (B) ACG (means ± SEM, N = 6). The black circles correspond to the 2P-reference rhodamine B in methanol and data were taken from the literature [22]. Ca^2+^-free ([Ca^2+^]_free_  = 0 nM) and Ca^2+^-saturated ([Ca^2+^]_free_  = 40 µM) conditions are depicted by blue and red squares, respectively. (C) Double logarithmic plot of the measured fluorescence intensity *I*_F_ as a function of 2P-excitation power *P* for rhodamine B (black circles) in methanol, ACR (red squares) and ACG (green triangles) in a Ca^2+^-saturated buffer (λ_ex,2P_  = 780 nm; N = 3). The data points were fitted by a linear function (*r^2^*≥0.98).

With regard to biological applications, 2P-excitation offers several advantages in comparison to 1P-excitation, such as deeper tissue penetration, less light scattering, lower cell toxicity and autofluorescence [30]. To choose optimal excitation properties, the knowledge of 2P fluorescence excitation spectra is necessary. The efficiency of the 2P-excitation process can be quantified by the fluorescence excitation action cross-section, which is the product of fluorescence quantum yield Φ_F_ and 2P-absorption cross-section σ_2_. The latter was determined by relative measurements, whereby rhodamine B in methanol as well as the characterized 2P-reference was used [22], [23]. The fluorescence quantum yield of rhodamine B (Φ_F_ = 0.50±0.003; N = 5) was measured absolutely and is in good agreement with the published data [31]. By means of absorption spectra, the dye concentrations were determined more accurately [32]. In the range of λ_ex,2P_ = 720 nm–860 nm the obtained 2P-absorption cross-sections σ_2_ of ACR were rather constant, and only increased slightly at longer wavelengths σ_2_ (Figure 2A). Moreover, the obtained σ_2_ values for Ca^2+^-free and Ca^2+^-bound ACR were relatively small compared to other long excitation wavelength Ca^2+^-sensitive dyes and 2P-references rhodamine B or fluorescein [22], [23], [33]. However, even dyes with small 2P-action cross-sections can be successfully applied in 2P-experiments, as we have previously shown for the Cl^-^ sensor MQAE and the Na^+^ sensor SBFI, exhibiting relatively small Φ_F_σ_2_ values <1 GM [17], [34]. For ACR, the calculated Φ_F_σ_2_ values for the Ca^2+^-bound species were always around one order of magnitude higher than that of the Ca^2+^-free species at λ_ex,2P_ = 780 nm, the wavelength used for 2P-FLIM *in situ* recordings; Φ_F_σ_2_ values of 0.04 GM (Ca^2+^-free) and 1.38 GM (Ca^2+^-bound) were determined (Figure 2A). So, an increase in 2P-excited fluorescence intensity due to increasing [Ca^2+^]_free_ is expected. Interestingly, recently published relative 2P-excited fluorescence spectra recorded in neuronal cells showed a different trend, with a maximum shifted to below 800 nm [16].

ACG showed a local maximum of Φ_F_σ_2_ at λ_ex,2P_ = 820 nm for the Ca^2+^-free as well as Ca^2+^-bound species and continues to increase at longer wavelengths (Figure 2B). For the Ca^2+^-bound ACG species, higher Φ_F_σ_2_ values were determined in comparison to ACR. Thus, the Φ_F_σ_2_ values of the Ca^2+^-bound ACG species were in the same range as those of rhodamine B and thus slightly higher than those of other commercially available Ca^2+^ fluorophors like OGB-1 [21]. Similar to ACR, the calculated Φ_F_σ_2_ values for the Ca^2+^-bound species were always higher than those of the Ca^2+^-free species, and at λ_ex,2P_ = 780 nm, Φ_F_σ_2_ values of 0.15 GM and 44.55 GM were determined (Figure 2B). Thus, a rise in the 2P-excited fluorescence intensity due to increasing [Ca^2+^]_free_ is expected.

2P-excitation is a non-linear process. In this case, the measured fluorescence intensity *I*_F_ is a quadratic function of the excitation power, so in terms of ideal conditions the slope in a double-logarithmic plot is 2 [35]. This coincided for ACR (slope  = 2.0±0.1) and ACG (slope  = 2.0±0.1), and a slightly lower slope was calculated for rhodamine B (slope  = 1.6±0.1). Nevertheless, in all cases, a 2P-absorption process could be assumed (Figure 2C).

### *In vitro* time-resolved fluorescence measurements

Concerning quantitative [Ca^2+^]_i_ recordings in living cells, time-resolved fluorescence measurements are an alternative approach to well-established fluorescence intensity measurements as has already been shown [5], [6], [13], [21]. In contrast to the latter, the fluorescence decay time is mostly independent of dye concentration, so that problems like dye leakage and dye bleaching are circumvented [36]. The suitability of ACR and ACG for time-resolved fluorescence measurements was tested *in vitro* by using the commercial Calcium Calibration Buffer Kit #1. Therefore, 11 solutions of varying [Ca^2+^]_free_ were prepared, and subsequently time-resolved fluorescence measurements were carried out. Both dyes were 2P-excited at 780 nm and the emitted fluorescence was recorded using the TCSPC technique. Theoretically, the easiest assumption would be the formation of 1∶1 complexes between Ca^2+^ ions and the Ca^2+^-sensitive dye. Hence, two distinct fluorescence decay time components would be expected, corresponding to the Ca^2+^-free and Ca^2+^-bound dye forms, respectively. Only their fractions relative to the entire fluorescence decay curve will change with varying [Ca^2+^]_free_.

**Figure 3:**
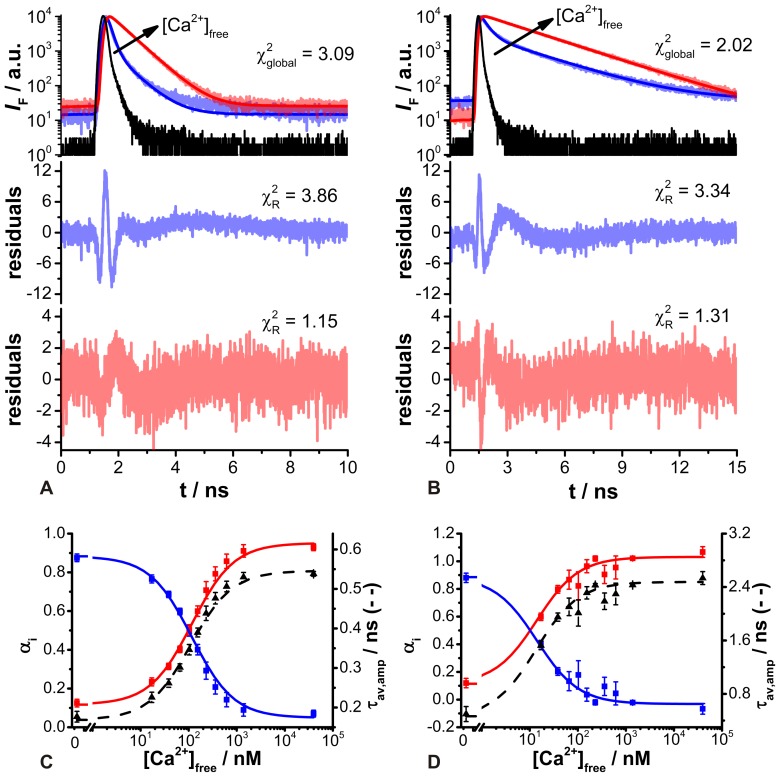
Time-resolved fluorescence recordings of ACR and ACG after 2P-excitation at 780 nm. Fluorescence decay curves of (A) ACR and (B) ACG in Ca^2+^-free (blue, [Ca^2+^]_free_  = 0 nM) and Ca^2+^-saturated (red, [Ca^2+^]_free_  = 40 µM) buffer solutions and the corresponding global biexponential deconvolution fits with 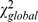 values; IRF: instrument response function (black). The black arrow indicates increasing [Ca^2+^]_free_. The residuals and individual 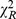 values of the global biexponential fits are shown below. Normalized amplitudes α_i_ (squares, solid lines) and amplitude-weighted average fluorescence decay time τ_av,amp_ (triangles, dashed line) as a function of [Ca^2+^]_free_ for (C) ACR and (D) ACG. The blue squares correspond to the normalized amplitudes of the short decay time component (ACR: 0.12 ns±0.006 ns and ACG: 0.25 ns±0.04 ns; Ca^2+^-free species), whereas the normalized amplitudes of the long decay time component (ACR: 0.57 ns±0.003 ns and ACG: 2.38 ns±0.02 ns; Ca^2+^-bound) are depicted by red squares, (means ± SEM, N = 5).

The obtained fluorescence decay curves of the Ca^2+^ concentration series for ACR and ACG showed a complex decay behavior and were globally fitted by a biexponential deconvolution fitting routine, in which the two decay time components were linked. The fits resulted in reasonable 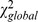 values and uniformly alternating residuals. Only around the fluorescence peak maximum were larger deviations observed (Figure 3A–3B). This could hint at an additional decay time component. However, a triexponential fitting model yielded slightly improved 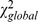 values and residuals (ACR: 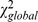 3.09 *vs.* 2.20 and ACG: 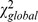 2.03 *vs.* 1.60). The third decay time component for both dyes was calculated at approximately 20 ps–30 ps. These short decay times were considered uncertain, since such short decay times cannot be reliably resolved with the present setup displaying an IRF of 220 ps (FWHM). Thus, ACR exhibited two distinct fluorescence decay time components most probably attributed to the Ca^2+^-free (τ_free_ = 0.12 ns±0.006 ns) and the Ca^2+^-bound species (τ_bound_ = 0.57 ns±0.003 ns), respectively. For ACG, the decay time component of the Ca^2+^-free form is in the same range as that for ACR (τ_free_ = 0.25 ns±0.04 ns). On the other hand, the decay time component of the Ca^2+^-bound form is considerably higher (τ_bound_ = 2.38 ns±0.02 ns). The stated errors of the calculated fluorescence decay times were fitting errors derived from the FAST fitting routine.

For both dyes, the normalized amplitudes α_i_ showed an opposite trend due to changes in [Ca^2+^]_free_. Thereby, the normalized amplitudes α_f_ of the short decay time component, corresponding to the Ca^2+^-free dye form, decreased when [Ca^2+^]_free_ increased, whereas the normalized amplitudes α_b_ of the long decay time component, corresponding to the Ca^2+^-bound dye form, rose due to increasing [Ca^2+^]_free_ (Figure 3C–3D). It is somehow surprising that the normalized amplitudes α_i_ did not reach the initial fractions one and zero in Ca^2+^-saturated and Ca^2+^-free buffers, as we expected to find only the ion-free dye form in the Ca^2+^-free buffer and the ion-bound dye form of ACR and ACG in the Ca^2+^-saturated buffer. This phenomenon has been already discussed for other Ca^2+^-sensitive dyes and was either attributed to dye-aggregates or dye-impurities [13], [14], [21]. Nevertheless, a final explanation has not yet been confirmed.

Moreover, the determined fitting parameters allowed the calculation of the amplitude-weighted average decay time τ_av,amp_. Hence, in both cases τ_av,amp_ increased with rising [Ca^2+^]_free_. Fitting the obtained data by means of Equation (3) resulted in corresponding *K*_D,t_ values (Figure 3C–3D). So, in Ca^2+^ calibration buffer solutions, ACR and ACG exhibited *K*_D,t_ values of 114 nM±11 nM and 15 nM±3 nM, respectively. These dissociation constants obtained from time-resolved fluorescence measurements *K*_D,t_ deviate considerably from the dissociation constants determined from steady-state fluorescence measurements *K*_D,i_. Similar characteristics have been described previously and a correlation between *K*_D,i_ and *K*_D,t_ was given by Equation (4) [6], [37], [38], [39].
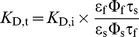
(4)


The subscripts *f* and *s* correspond to Ca^2+^-free and Ca^2+^-saturated conditions, respectively. In the case of 2P-excitation, the molar absorption coefficient ε needed to be replaced by its 2P analog, the 2P-absorption cross-section σ_2_, resulting in Equation (5).
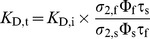
(5)


Thus, it is possible to estimate *K*_D,t_ for ACR and ACG from steady-state fluorescence measurements (see above) to approximately 60 nM±5 nM and 9 nM±3 nM, respectively. The calculated *K*_D,t_ values for both dyes are slightly lower than the measured one, but they are within the same order of magnitude. In general, the dissociation constant *K*_D_ indicates the measurable analyte concentration range from approximately 0.1 to 10 times the *K*_D_. However, the apparent value of *K*_D_ is determined by the particular measured parameter as it has been shown, for instance, for other Ca^2+^ indicator dyes recording fluorescence intensities or fluorescence decay times [6], [37]. Thus, when altering the measured parameter of an indicator dye, its dynamic range could probably be extended.

### *In vitro* steady-state and time-resolved fluorescence measurements of ACR in the presence of BSA

**Figure 4:**
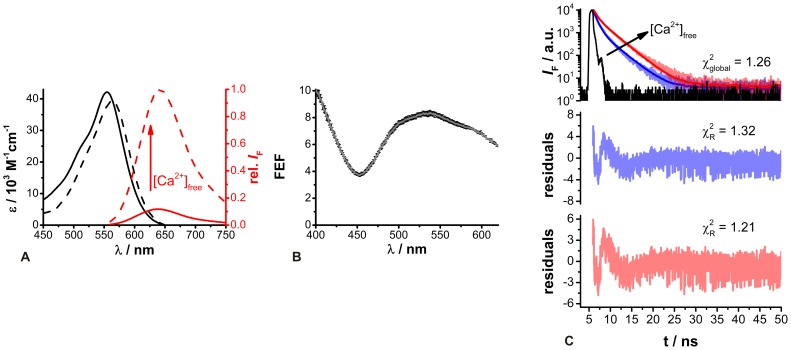
Spectroscopic properties of ACR in 1% BSA *in vitro* buffer solutions. (A) Absorption (black) and relative fluorescence (red) spectra of ACR (c = 2.5 µM, λ_ex_  = 550 nm) under Ca^2+^-free (solid lines) and Ca^2+^-saturated (dashed lines) conditions. (B) Fluorescence enhancement factor (FEF) of ACR as a function of excitation wavelength (means ± SEM, N = 3). The FEF is the ratio of the fluorescence intensities of the excitation spectra under Ca^2+^-saturated and Ca^2+^-free conditions. (C) Fluorescence decay curves of ACR in Ca^2+^-free (blue) and Ca^2+^-saturated (red) buffers (λ_ex_ = 550 nm, 1P-excitation, λ_em_ = 640 nm) and the corresponding global biexponential deconvolution fits with the 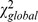 value; IRF: instrument response function (black). The black arrow indicates increasing [Ca^2+^]_free_. The residuals and individual 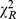 values of the global biexponential fits are shown below.

Concerning intracellular ion recordings, the spectroscopic parameters of ion-sensitive fluorescent dyes can be changed due to factors such as viscosity, pH and ionic strength demanding fluorescence measurements under cellular conditions. Due to manifold factors, which are additionally often incalculable, the comprehensive analysis of interactions between a dye and cellular compounds would require extensive studies. Since particularly the presence of proteins significantly influences dyes, the protein-dye interaction can be initially investigated by using BSA [40], [41], [42]. The presence of BSA (1% (*m/v*)) in the *in vitro* buffer solutions resulted in a bathochromic shift in the Ca^2+^-free ACR absorption maximum from 537 nm to 555 nm, and increasing [Ca^2+^]_free_ led to a further bathochromic shift from 555 nm to 563 nm (Figure 4A). Moreover, the molar absorption coefficients were also decreased by about 20% compared to those measured in the BSA-free *in vitro* calibration buffer.

The fluorescence intensity of ACR under Ca^2+^-free conditions was increased in the presence of BSA and rose upon [Ca^2+^]_free_ elevation. However, the fluorescence spectrum exhibited a hypsochromic shift from 654 nm to 641 nm (Figure 4A). In the presence of BSA, the maximum FEF was achieved at λ_ex_ = 538 nm with a FEF ∼8, which was about 4 times lower than that in the absence of BSA (Figure 4B *vs.* Figure 1E). This behavior could be also revealed by the absolutely determined fluorescence quantum yields Φ_F_ = 0.01 (N = 3) for the Ca^2+^-free (λ_ex_ = 543 nm) and Φ_F_ = 0.08 (N = 3) for the Ca^2+^-bound (λ_ex_ = 550 nm) ACR species in 1% BSA *in vitro* buffer solutions. Obviously, the influence of BSA seemed to be stronger on the Ca^2+^-free than on the Ca^2+^-bound ACR species. In fact, the bathochromic shift in the absorption spectrum and the hypsochromic shift in the emission spectrum as well as enhanced intensity have also been reported for other Ca^2+^-sensitive dyes such as Fura-2, Indo-1 and Quin-2 [41], [42], [43]. Since the fluorescent backbones of these dyes are different, it was suggested that the carboxyl groups of the dye's Ca^2+^-chelating side interact with proteins. Due to their higher affinity to Ca^2+^ ions the carboxyl group would be rather occupied by Ca^2+^ than by proteins. Thus, in the presence of Ca^2+^ the formation of a protein-dye complex is interfered.

The obtained fluorescence decay curves could be adequately fitted by a deconvolution biexponential fitting routine yielding reasonable residuals and 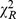 values (Figure 4C). The determined fluorescence decay time components for the Ca^2+^-free and the Ca^2+^-bound species were about 5 times longer than those obtained in BSA-free environments and amounted to τ_free_ = 0.77±0.002 ns (N = 3) and τ_bound_ = 2.86±0.04 ns (N = 3), respectively.

The 2P-absorption cross-sections of ACR in the presence of BSA were determined at λ_ex,2P_ = 780 nm as described previously, and yielded σ_2_ = 40 GM±1 GM (N = 3) for the Ca^2+^-free and σ_2_ = 24 GM ±1 GM (N = 3) for the Ca^2+^-bound dye species. Nevertheless, the 2P-action cross-section Φ_F_σ_2_ of the Ca^2+^-free and Ca^2+^-bound dye species were calculated to 0.4 GM and 1.9 GM, still indicating an increase in the ACR fluorescence intensity due to increasing [Ca^2+^]_free_ in the presence of BSA. All spectroscopic parameters determined in BSA-containing buffer solutions deviated from those perceived in BSA-free calibration buffer solutions, which can be attributed to dye-protein interactions. This fact underlines the need for determining the calibration parameters used for intracellular ion concentration quantification individually in each biological system.

### Time-resolved fluorescence measurements of ACR *in situ*

**Figure 5:**
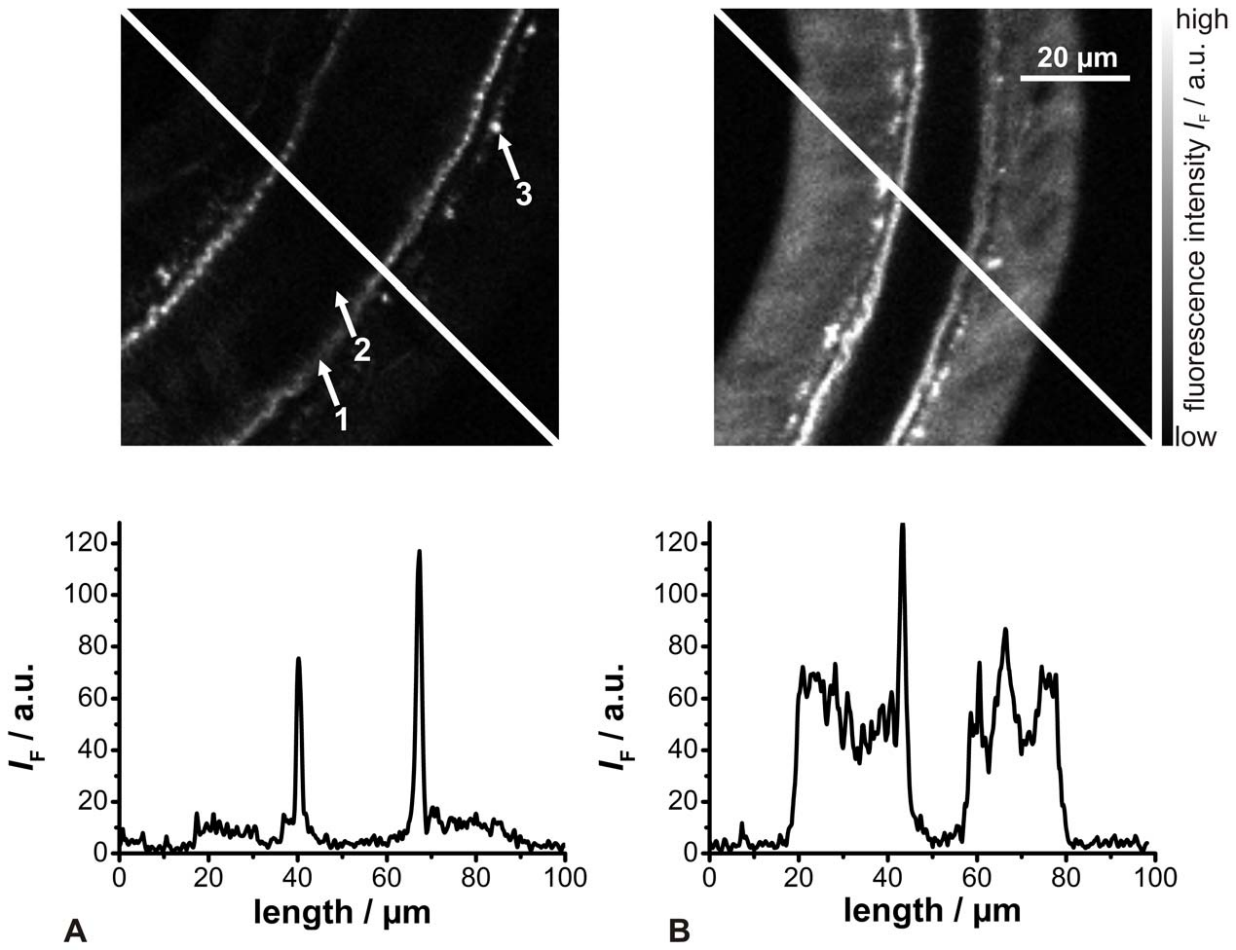
Behavior of ACR in cockroach salivary glands. 2P-excited (780 nm) fluorescence intensity images of (A) unloaded (1 luminal cuticule, 2 ductal lumen, 3 apically located, point-shaped structures) and (B) ACR-loaded salivary gland ducts (median optical sections). The graphs below the images display the fluorescence intensity traces along the white lines in the images.

Although the *in vitro* measurements of ACG have unraveled larger absorption coefficients, higher fluorescence quantum yields, and larger FEFs compared to those of ACR, at this time ACG is still only commercially available in K^+^-salt form, but not in the ester form required for experiments in living tissues. Indeed, the ACG salt form could be microinjected into a selected living cell for *in situ* recordings. However, this would be an invasive approach and the simultaneous analysis of several cells would be virtually impossible. On the other hand, ACR is commercially available as an AM-ester. So, subsequently the feasibility of ACR for 2P-FLIM recordings in living cells was tested. The investigated salivary gland ducts showed a relatively low cellular autofluorescence after 2P-excitation at λ_ex,2P_  = 780 nm, but with a distinct signal induced by the luminal cuticle (Figure 5A). In contrast, the fluorescence intensity of the duct cells was increased nearly 6 times after ACR loading (Figure 5B). Moreover, an inhomogeneous dye loading in terms of significantly weaker nuclei staining was observed. The same behavior has recently been described for the Ca^2+^ sensor OGB-1 [21].

Furthermore, the influences of dye leakage and dye bleaching of ACR after 2P-excitation were determined, as both effects are limiting factors for long-term measurements. Therefore, fluorescence intensity images were recorded for 60 min at a low acquisition rate of 0.067 min^−1^ (*P* = 3.3 mW, measured at objective aperture) and for 30 min at an increased acquisition rate of 1.3 min^−1^ (*P* = 3.7 mW). In the first case, the initial fluorescence intensity is reduced by one-third after 60 min, which can presumably be attributed to dye leakage (Figure S2A). This is in a comparable range to other Ca^2+^-sensitive dyes [21]. In the second case, the recorded fluorescence intensity showed a gradual decrease and was fitted by a monoexponential decay function, which yielded a half-time *t_½_* of fluorescence dye bleaching of about *t*_½_ = 14 min (Figure S2B). Compared to other Ca^2+^-sensitive dyes such as OGB-1, the *t_½_* of ACR is almost two times shorter [21]. This fact has to be taken into account for long-term measurements, especially for fluorescence intensity-based ones.

**Figure 6:**
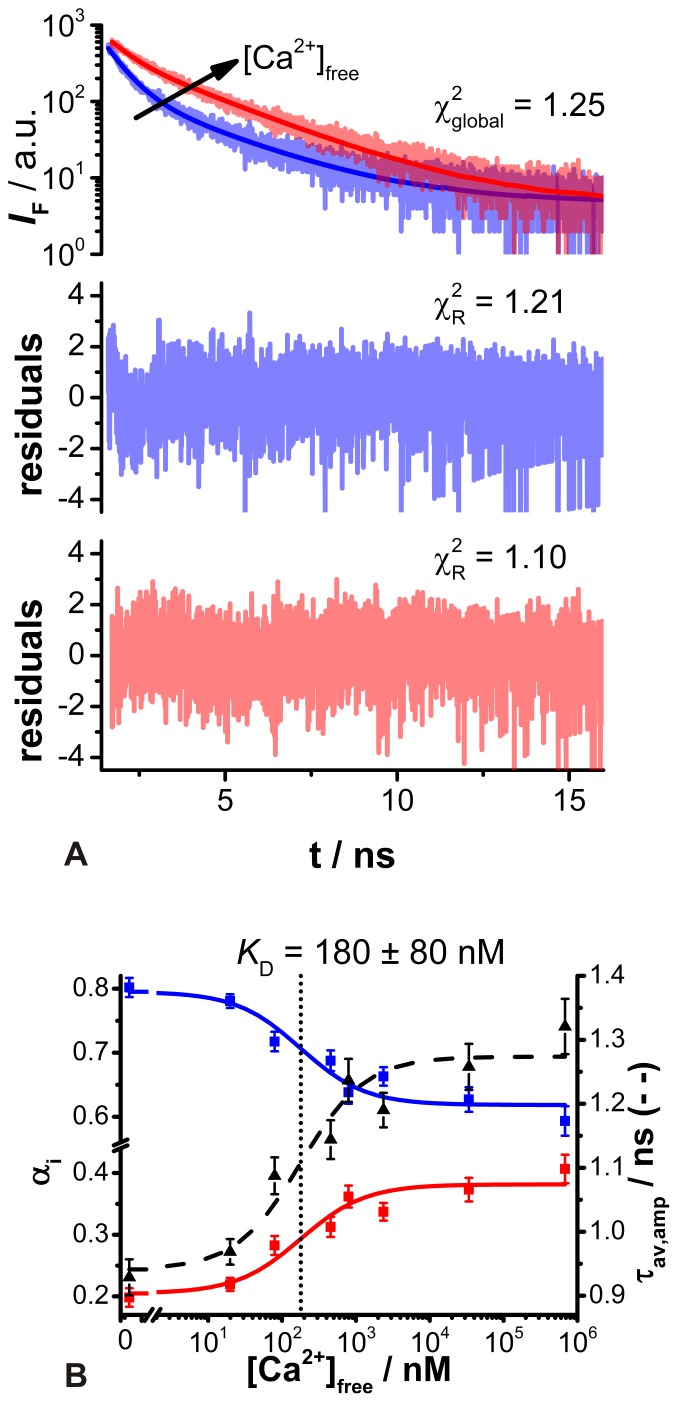
Determination of *in situ K*_D,t_ for ACR in salivary duct cells. (A) Fluorescence decay curves extracted from 2P-FLIM images of ACR-loaded salivary duct cells under Ca^2+^-free (blue, [Ca^2+^]_free_  = 0 nM) and Ca^2+^-saturated (red, [Ca^2+^]_free_  = 68 µM) conditions and the corresponding global biexponential deconvolution fits with the 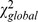 value. The residuals and individual 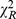 values of the global biexponential fits are shown below. (B) Normalized amplitudes α_i_ (squares, solid lines) and amplitude-weighted average fluorescence decay time τ_av,amp_ (triangles, dashed line). The blue squares correspond to the normalized amplitudes of the short decay time component (0.56 ns, Ca^2+^-free species), whereas the normalized amplitudes of the long decay time component (2.44 ns, Ca^2+^-bound species) are depicted by red squares (N = 11–23). The dotted line marks the determined *K*_D,t_.



As already shown for BSA, the spectroscopic properties of ACR are influenced by their environment. Additionally, several studies have shown that *in vitro* dissociation constants *K*_D_ differ from *in situ K_D_* [21], [44]. Due to this, the *K*_D_ value has to be determined individually for each experimental setup and biological system. Thus, *in situ* calibration experiments were performed using the non-ionic surfactant Triton X-100 as an equilibrating agent [21]. Hence, the salivary glands were continuously perfused with a calibration buffer solution containing defined [Ca^2+^]_free_ and 0.1% Triton X-100. This Triton X-100 concentration enabled a [Ca^2+^]_free_ equilibration without significant intracellular dye loss and tissue deterioration within the experimental time period in the cockroach salivary glands, as reported recently [21]. Every minute a 2P-FLIM image was recorded, and for data analysis images were taken with the maximum Ca^2+^ effect, but without visible tissue deformation. Thereby, 126 fluorescence decay curves were obtained from ROIs, which were fitted globally by the biexponential deconvolution function, yielding reasonable residuals and 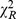 values (Figure 6A). Thus, two distinct decay time components were identified at τ = 0.56 ns and τ = 2.44 ns, corresponding most probably to Ca^2+^-free and Ca^2+^-bound ACR, respectively. These time components were in the same range as those measured in the BSA-buffer, indicating a protein-dye interaction causing longer fluorescence decay times. The obtained fitting parameters α_i_ and τ_av,amp_ were plotted against corresponding [Ca^2+^]_free_ and fitted by Equation (3), unraveling the *in situ* dissociation constant of ACR to *K*_D,t_ = 180 nM±80 nM (Figure 6B). This is about 1.7 times larger than the measured *in vitro K*_D,t_ value. The only available *in situ K*_D_ values for ACR so far were published by Ljubojević et al. [44]. In that study, *K*_D,i_ values were determined in rat ventricular cardiomyocytes at 2183 nM in nucleoplasm and 1336 nM in cytoplasm, respectively. However, both *K*_D_ values are not directly comparable to the values presented here, since they are based on fluorescence intensity-based measurements which seem to result in larger *K*_D_ values. The measurable analyte concentration range is thought to be between 0.1 and 10 times that of the *K*_D_ [7]. Thus, the determined *in situ K*_D,t_ of ACR fits well with the expected [Ca^2+^]_i_ changes in cockroach salivary gland duct cells [21], [45]. On the other hand, the changes in τ_av,amp_ were unfortunately very small, so quantification of low [Ca^2+^]_i_ changes seems to be challenging (Figure 6B). Conversion of the effective amplitude-weighted average fluorescence decay time τ_av,amp_ into [Ca^2+^]_i_ has been suggested previously according to Equation (6)

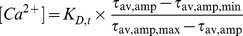
(6)with the extreme τ_av,amp_ values at Ca^2+^-free (τ_av,amp,min_) and Ca^2+^-saturated (τ_av,amp,max_) conditions, respectively [38], [37].

**Figure 7:**
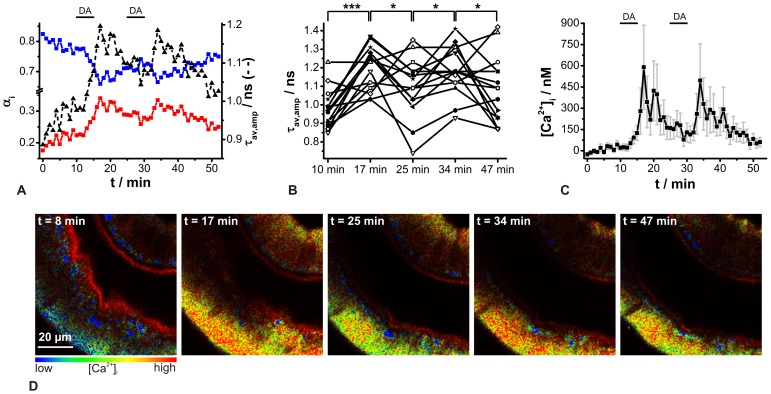
Analysis of dopamine-induced [Ca^2+^]_i_ changes in ACR-loaded salivary duct cells by 2P-FLIM recordings. (A) Normalized amplitudes α_i_ (squares, solid lines) and amplitude-weighted average fluorescence decay time τ_av,amp_ (triangles, dashed line). The blue squares correspond to the normalized amplitudes of the short decay time component (0.56 ns, Ca^2+^-free species), whereas the normalized amplitudes of the long decay time component (2.44 ns, Ca^2+^-bound species) are depicted by red squares (means, N = 15). Black bars indicate the periods of 1 µM dopamine presence. (B) Statistical analysis of the dopamine effect; repeated-measures ANOVA and Holm-Sidak's multiple comparison tests (* *P*<0.05, *** *P*<0.001). (C) Data converted to [Ca^2+^]_i_. The error bars shown were calculated by error propagation of τ_av,amp_. Black bars indicate the periods of dopamine presence and (D) corresponding false color-coded 2P-FLIM images at distinct time points.

The feasibility of ACR for *in situ* measurements was tested in duct cells of cockroach salivary glands, representing a well-established model system for investigating aminergic controlled transepithelial ion transport processes [18]. The biogenic amine dopamine induces a saliva secretion accompanied by a reversible, slow, almost tonic, dose-dependent increase in [Ca^2+^]_i_ in the duct cells from 48 nM±4 nM to 311 nM±43 nM as demonstrated by steady-state fluorescence recordings using Fura-2 [45], [46]. A second, subsequent dopamine stimulus can yield an even four-times larger [Ca^2+^]_i_ elevation [45]. Recently, a dopamine-induced [Ca^2+^]_i_ increase from 144 nM±15 nM to 372 nM±160 nM was confirmed using the Ca^2+^ sensor dye OGB-1 for 2P-FLIM recordings [21]. Thus, this Ca^2+^ signaling response is a valuable tool for testing new Ca^2+^ sensor dyes *in situ*. When [Ca^2+^]_i_ increases, the corresponding ACR τ_av,amp_ should increase as well. To verify this assumption, ACR-loaded salivary duct cells were stimulated twice with 1 µM dopamine, and 2P-FLIM images were recorded continually (image acquisition time ∼30 s, acquisition rate 1 image min^−1^). The appropriate fluorescence decay curves were fitted by a biexpontential decay function with fixed decay time components from the *in situ* calibration. Based on the fitting results, the normalized amplitudes α_i_ as well as τ_av,amp_ values were calculated (Figure 7A). As ACR bleached quickly under the chosen conditions (see also above), the quality of the fluorescence decay curves deteriorated during the experiment. Nevertheless, a sufficient fluorescence signal was obtained until the end of a measurement, indicated by reasonable residuals and 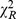 values. After a bath application of dopamine, the normalized amplitudes showed an opposite trend as expected and τ_av,amp_ rose statistically significantly and recovered to resting state level after dopamine washout (Figure 7A–7B). The second dopamine stimulus proved the reversibility of this process, as well as the viability of the tissue under these 2P-FLIM experimental conditions. By means of the *in situ* calibration, it was possible to correlate τ_av,amp_ values with quantitative [Ca^2+^]_i_ changes. Thereby, resting [Ca^2+^]_i_ was calculated to 21 nM±18 nM (N = 15). In the presence of dopamine, [Ca^2+^]_i_ increased to 590 nM±300 nM (N = 15). The second dopamine stimulus resulted in a similar rise to 500 nM±260 nM (N = 15) (Figure 7C). The described behavior could also be identified by false color-coded 2P-FLIM images, in which warmer colors correspond to higher [Ca^2+^]_i_ (Figure 7D). The obtained results fit qualitatively well to the previously published data using Fura-2 and OGB-1 [21], [45]. However, large variations of τ_av,amp_ values could be recognized between the individual experiments, causing broader calcium concentration distributions (Figure 7B–7C). A reason for this could be found in the spatio-temporal behavior of the dopamine-induced [Ca^2+^]_i_ rise. At first, in addition to a tonic [Ca^2+^]_i_ increase during dopamine application, some salivary gland preparations exhibit a slow decrease after reaching a [Ca^2+^]_i_ peak value. It is also known that the [Ca^2+^]_i_ rise starts at several cells and spreads slowly over the duct epithelium, but not all regions of the duct seem to respond [45]. Thus, depending on the relatively small duct region investigated (80 µm×80 µm), variable responses to dopamine could be expected. In addition, the image acquisition rate was limited to 1 min^−1^, making recordings of [Ca^2+^]_i_ rises of different magnitude also more likely. So, faster Ca^2+^ imaging could be realized by recording smaller regions of interest or directly at one single point. But then, a higher impact of photobleaching and tissue damage has to be taken into consideration. Another source of errors could be the weak fluorescence of the Ca^2+^-free ACR species. So, contribution of cellular autofluorescence to the overall fluorescence decay is increasingly strengthened by decreasing [Ca^2+^]_i_ and consequently might no longer be negligible. Evidence can therefore be seen in the resting [Ca^2+^]_i_, just alternating around zero. Another aspect which must be considered is the possible impact of varying intracellular pH. For ACR, it has been reported that protonation due to acidification leads to a fluorescence intensity increase in the Ca^2+^-free species without effecting the Ca^2+^-bound species [16]. Thus, a [Ca^2+^]_i_ rise accompanied by intracellular acidification could probably be detected with ACR at a higher [Ca^2+^]_i_ level than actually occurs. From the literature it is known that cockroach salivary gland duct cells acidify after dopamine stimulation, from about pH 7.3 to pH 6.9 [47]. This acidification would lead to an increase in the signal of the Ca^2+^-free ACR species of about 10%, which might cause a slight overestimation of the [Ca^2+^]_i_ rise.

## Conclusions

ACG and ACR are novel developed Ca^2+^-sensitive fluorescent dyes. Both dyes absorbed and emitted in the visible spectral range, whereas the fluorescence emission band of ACR is considerably red-shifted due to a large Stokes shift. Moreover, ACR exhibited a Ca^2+^-sensitive and an almost Ca^2+^-insensitive emission band and therefore could be used as emission-ratiometric dye. This is of special interest in terms of Ca^2+^ concentration quantification in living tissue. In contrast, ACG is a single-wavelength dye, which showed an almost 6-times higher FEF compared to ACR. Based on the determined dissociation constants *K*_D_, it can be concluded that both dyes are high-affinity Ca^2+^ indicators. ACR and ACG exhibited a change in the fluorescence quantum yield upon Ca^2+^ binding and thus met the requirement for time-resolved fluorescence measurements. Indeed, FLIM is a unique and versatile microscopic technique for quantitative analyte concentration measurements. In combination with the advantages of 2P-excitation, 2P-FLIM is especially suited for live cell imaging. Thus, the evaluation of new fluorescent dyes suitable for 2P-FLIM will further extend its field of application. Here, we showed for the first time the potential of ACR and ACG for time-resolved fluorescence measurements. Since ACR and ACG also showed sufficient 2P fluorescence excitation, these dyes were predestinated for 2P-FLIM application. Thereby, ACR as well as ACG showed a biexponential fluorescence decay behavior *in vitro* and a Ca^2+^-dependent change in their amplitude-weighted average fluorescence decay time τ_av,amp_. However, up to now ACG has not been available in the ester form required for experiments with living tissues. Also in living cells, ACR exhibited biexponential fluorescence decay behavior, which could be successfully converted into absolute [Ca^2+^]_i_. Therefore, *in situ* calibration is required in the particular biological system, since initial experiments of ACR with BSA *in vitro* have already unraveled possible protein-dye interactions. The determined properties of ACR and ACG were summed up in Table 1 together with data for the well-established Ca^2+^ indicator OGB-1, which has already been used for 2P-FLIM in living cells [13], [21]. In summary, ACR is a quite promising new Ca^2+^ indicator for 2P-FLIM recordings in diverse biological systems. Since ACR exhibits a prominent, large Stokes shift, it is also a promising candidate for multiplexing approaches to analyzing the spatio-temporal behavior of several physiological parameters at the same time. Thereby, after simultaneous 2P-excitation of several sensor dyes, their fluorescence signals could be distinguished by spectral separation and/or fluorescence decay times.

**Table 1:** Summary of determined parameters of ACR and ACG in comparison to those of OGB-1.

		ACR	ACG	OGB-1
Absorption maximum	λ_f_/nm	537	515	494 a
	λ_s_/nm	542 (540) b	519 (518) c	494 a
Molar absorption coefficient	ε_f_/10^3^ M^-1^cm^−1^	55±1.3 (56±0.5) b	130±2.0	76 d
	ε_s_/10^3^ M^-1^cm^−1^	47.8±0.8 (50±0.9) b	144±1.6	78 d
Emission maximum	λ_f_/nm	654	542	523 d
	λ_s_/nm	654 (650) b	542 (540) c	523 d
Maximum fluorescence enhancement factor	FEF	∼41* (35–45) ^1b^	∼213** (220) ^1c^	∼14 d
Fluorescence quantum yield	Φ_F,f_	0.0017 e	0.0020 e (0.0023) c	0.06±0.01 a
	Φ_F,s_	0.06±0.01	0.42±0.01 (0.495) c	0.75±0.01 a
2P fluorescence excitation action cross sections at λ_ex,2P_ = 780 nm	Φ_F_σ_2,f_/GM	0.04	0.15	3 a
	Φ_F_σ_2,s_/GM	1.38	44.55	26 a
Dissociation constant from steady-state fluorescence (*in vitro*)	*K*_D,i_/nM	463±59 (490±70) b	265±33 (135) c	170 d
Ratiometric dye		yes	no	no d
Fluorescence decay time components (*in vitro*)	τ_f_/ns	0.12±0.006	0.25±0.04	0.53 a
	τ_s_/ns	0.57±0.003	2.38±0.02	3.73 a
Dissociation constant from time-resolved fluorescence (*in vitro*)	*K*_D,t_/nM	114±11	15±3	410±30 a
AM-ester form		yes	no	yes
Fluorescence decay time components (*in situ*)	τ_f_/ns	0.56	n/a	0.62 a
	τ_s_/ns	2.44	n/a	3.45 a
Dissociation constant from time-resolved fluorescence (*in situ*)	*K*_D,t_/nM	180±80	n/a	640±180 a

The subscripts *f* and *s* correspond to Ca^2+^-free and Ca^2+^-saturated buffer conditions, respectively.

*λ_ex_  = 548 nm.

**λ_ex_  = 525 nm.

a Data from [21].

b Data from [16]. ^1b^Data from [16] (λ_ex_  = 540 nm).

c Data from [29]. ^1c^ Data from [29] (λ_ex_  = 525 nm).

d Data from [48].

e As the absolute fluorescence quantum yield Φ_F_ of ACR and ACG in the Ca^2+^-free buffer were below the limit of detection, they were estimated relatively. Thus, as reference the absolute fluorescence quantum yields of ACR and ACG in Ca^2+^-saturated buffer were used [25].

## Supporting Information

Figure S1 **Steady-state fluorescence spectra of ACR at excitation wavelength λ_ex_ = 488 nm.** Fluorescence of ACR (c = 2.5 µM) was recorded in aqueous buffer solutions of varying [Ca^2+^]_free_ from 0 µM–40 µM. The *inset* shows the [Ca^2+^]_free_ dependent normalized fluorescence intensity at the emission wavelengths λ_em_ = 525 nm (blue triangles) and λ_em_ = 650 nm (red triangles).
(TIF)

Figure S2 **Leakage and photobleaching of ACR in salivary duct cells.** Salivary gland lobes were incubated with 5.1 µM ACR/AM for 60 min. After acclimatization, 2P-FLIM images were recorded at λ_2P-ex_ = 780 nm. (A) Low image acquisition rate (0.067 min^−1^, P = 3.3 mW) presumably indicates dye leakage from the cells (blue squares, means ± SEM, N = 5). Fit to monoexponential decay function yielded a leakage half-time of *t*_1/2_ = 61 min (dashed black curve). High image acquisition rate (1.3 min^−1^, P = 3.7 mW) presumably indicates dye photobleaching (red squares, means ± SEM, N = 4). Fit to monoexponential decay function yielded a photobleaching half-time of *t*_1/2_ = 14 min (solid black curve).
(TIF)
