# Supplementary figures and images for: Asante Calcium Green and Asante Calcium Red—Novel Calcium Indicators for Two-Photon Fluorescence Lifetime Imaging
Source: PLoS One. 2014 Aug 20;9(8):e105334. doi: 10.1371/journal.pone.0105334 (PMC4139374; doi:10.1371/journal.pone.0105334)

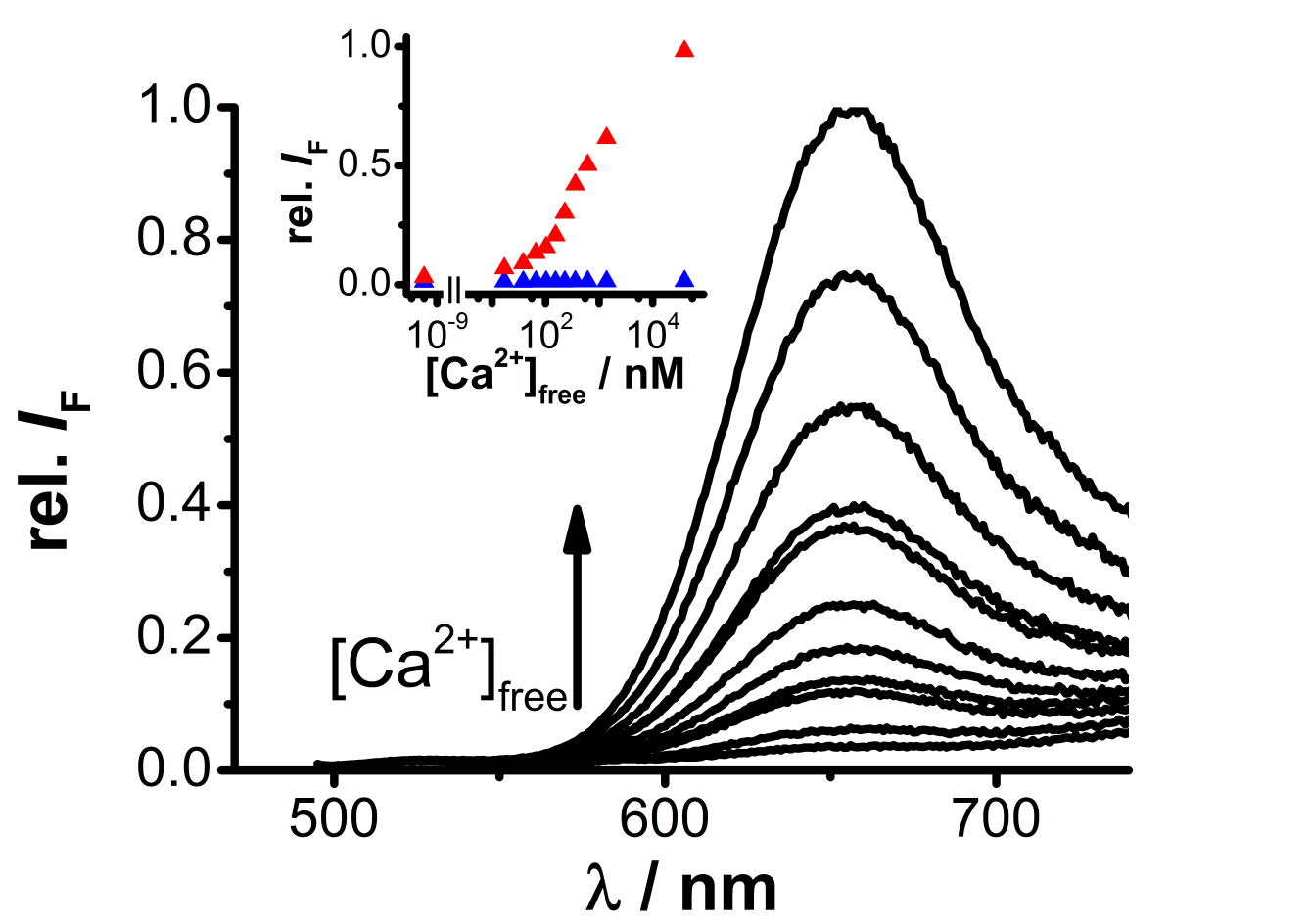

Supplement: Figure S1 — Steady-state fluorescence spectra of ACR at excitation wavelength λex = 488 nm. Fluorescence of ACR (c = 2.5 µM) was recorded in aqueous buffer solutions of varying [Ca2+]free from 0 µM–40 µM. The inset shows the [Ca2+]free dependent normalized fluorescence intensity at the emission wavelengths λem = 525 nm (blue triangles) and λem = 650 nm (red triangles). (TIF) [file pone.0105334.s001.tif]

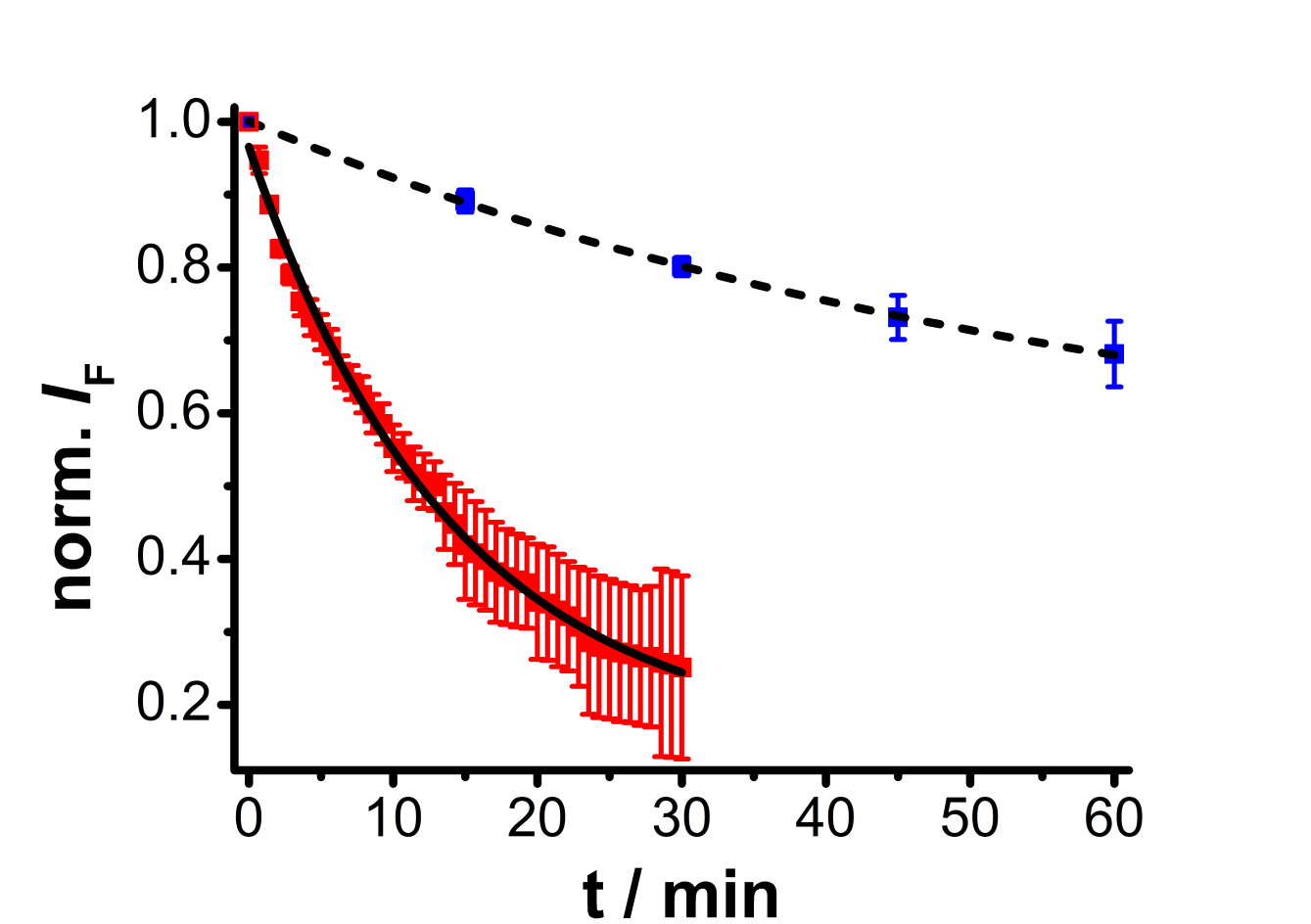

Supplement: Figure S2 — Leakage and photobleaching of ACR in salivary duct cells. Salivary gland lobes were incubated with 5.1 µM ACR/AM for 60 min. After acclimatization, 2P-FLIM images were recorded at λ2P-ex = 780 nm. (A) Low image acquisition rate (0.067 min−1, P = 3.3 mW) presumably indicates dye leakage from the cells (blue squares, means ± SEM, N = 5). Fit to monoexponential decay function yielded a leakage half-time of t 1/2 = 61 min (dashed black curve). High image acquisition rate (1.3 min−1, P = 3.7 mW) presumably indicates dye photobleaching (red squares, means ± SEM, N = 4). Fit to monoexponential decay function yielded a photobleaching half-time of t 1/2 = 14 min (solid black curve). (TIF) [file pone.0105334.s002.tif]
